# Deep time-delay Markov network for prediction and modeling the stress and emotions state transition

**DOI:** 10.1038/s41598-020-75155-w

**Published:** 2020-10-22

**Authors:** Barlian Henryranu Prasetio, Hiroki Tamura, Koichi Tanno

**Affiliations:** 1 Interdisciplinary Graduate School of Agriculture and Engineering, University of Miyazaki, Miyazaki, 889-2192 Japan; 2 Faculty of Engineering, University of Miyazaki, Miyazaki, 889-2192 Japan

**Keywords:** Biomedical engineering, Computer science, Information technology

## Abstract

To recognize stress and emotion, most of the existing methods only observe and analyze speech patterns from present-time features. However, an emotion (especially for stress) can change because it was triggered by an event while speaking. To address this issue, we propose a novel method for predicting stress and emotions by analyzing prior emotional states. We named this method the deep time-delay Markov network (DTMN). Structurally, the proposed DTMN contains a hidden Markov model (HMM) and a time-delay neural network (TDNN). We evaluated the effectiveness of the proposed DTMN by comparing it with several state transition methods in predicting an emotional state from time-series (sequences) speech data of the SUSAS dataset. The experimental results show that the proposed DTMN can accurately predict present emotional states by outperforming the baseline systems in terms of the prediction error rate (PER). We then modeled the emotional state transition using a finite Markov chain based on the prediction result. We also conducted an ablation experiment to observe the effect of different HMM values and TDNN parameters on the prediction result and the computational training time of the proposed DTMN.

## Introduction

Emotion plays a vital role in communication. Emotional awareness helps us to better understand the feelings of a communicator. In the 1970s, a psychologist identified six basic emotions: happiness, sadness, disgust, fear, surprise, and anger^1^. In human life, happiness is the primary purpose to be achieved. Happiness is often defined as a pleasant emotion. In contrast, unhappiness is projected to an unpleasant state, such as sadness, depression, and stress^2^. In neurobiology science, stress is a situation that triggers a particular biological response that causes hormones to surge throughout the body^3^. When people are in a stressed condition, it is easy for them to misunderstand intentions or what they would like to communicate and express an abnormal emotion as a reaction. Stress can affect all aspects of a person’s life, including emotions, behaviors, thinking ability, and physical health^4^. Everyone handles stress in different ways so that the symptoms of stress are also varied. The symptoms of stress can be vague and may be the same as other medical conditions. Hence, it is important to recognize stress early.

The body reveals the stress response through facial expression, body language, and tone of voice. Thus, the facial expression^5–8^ and speech of the stressed person^9–13^ can be used to detect the level of stress^14^. Since the speech-based stress measurement method is non-invasive, it is convenient for measuring stress. Therefore, this method has become popular and widely studied. The speech-based stress measurement method, also known as stress speech recognition (SSR), uses labeled utterances and learns their patterns to recognize stress^12^. A large quantity of relevant stress speech data is required in the training phase to enable this system to adapt to real conditions. Unfortunately, stress speech datasets are limited. To this end, many researchers use clustering algorithms to categorize unlabeled stressed speech data based on the similarity of their characteristics.

In this decade, clustering algorithms have successfully categorized stress speech data using an unsupervised approach^15–18^. Most of them used a similarity algorithm to compute the distance between data points. However, it was found that these algorithms become inefficient for high-dimensional data due to their computation time and memory usage^19^, known as the curse of dimensionality. Recently, using a self-learning method to optimize the clustering objective, deep clustering algorithms have addressed the curse of dimensionality^20^ problem. Deep clustering applies a deep neural network (DNN)-based autoencoder to compactly transform the data from the original space to a lower-dimensional space (embedding space)^21,22^. By learning in-depth and simultaneously minimizing the error, deep clustering can present an excellent feature representation. However, despite its compactness in representing features, most of the deep clustering algorithms have not yet considered the prior state. In some cases, emotion (especially stress) may change when triggered by an event while speaking^23^. In this fashion, we argue that the prior emotional states should also be monitored so that the emotion of the speaker can be recognized more accurately. By this approach, we can take advantage of larger sets of contextual information^24^.

Several studies have successfully modeled emotion based on its state transition^23–27^. Generally, for predictive modeling or probabilistic forecasting^28^, the Markov model is the most used because of its convenience in modeling the temporal context in time-series (continuous) data^27,29^. The hidden Markov model (HMM) models the dependencies between consecutive hidden states. In natural language processing, it was found that there are local dependencies and at a distance. Conservative methods that use the most recent history to perform prediction produce an overfitting result for short-term patterns and miss the important long-term effects^30^. Thus, capturing the long-term temporal dynamics in-depth is essential for further exploration.

Today, deep neural networks (DNNs) are the most popular deep learning technique because of their superior in-depth learning of complex patterns. DNNs are composed of multiple layers of nonlinear operations that aim to learn features hierarchically, where features in a small temporal context at higher layers are formed using the features at lower layers. To process a wider temporal context, from the initial layer, DNN learns an affine transform for the entire temporal context^31^. Consequently, DNNs become ineffective for modeling the dependencies of temporal dynamics (long and short temporal contexts)^32^, such as stressed speech^33^. In contrast, to handle a long-range temporal dependence, a time-delay neural network (TDNN) creates more large networks from sub-components across time steps^31^. In such a way, TDNN learns the dependency inter-contexts at small or long temporal scenarios.

To this end, we propose a new framework for predicting and modeling stress and emotions, named the deep time-delay Markov network (DTMN). The DTMN analyses in-depth the stress and emotion speech features by considering the prior emotional states. Structurally, the DTMN contains Markov method, which is handled by HMM and the neural network architecture of TDNN. HMM is trained to generate the transition matrix of emotional states and predict the hidden states at each time step. The TDNN is trained to predict the present hidden state by considering the present feature and prior hidden states. We explicitly use the embedding feature of deep clustering^22^ as input to the DTMN, which proves able to present a compact feature representation of stress and emotion.

We organized the rest of this paper as follows. In the “Related works” section, we review the existing stress and emotion models and the related works. The “Results” section demonstrates the evaluation results in the prediction task and the modeling of stress and emotion transitions. The prediction result and the state transition model of the stress and emotions are discussed in the “Discussion” section. The “Method” section describes the material and method of the proposed DTMN that consists of the use of the dataset, network settings, baseline systems, and its ablation experiment. Finally, the “Conclusion” section provides the final results and future work.

## Related works

In this decade, stress and emotion recognition systems using speech analysis have been extremely studied. Most of them used a standard architecture where the feature extraction and classifier were the main components in recognizing the stress and emotion patterns. The effectiveness of feature representation is a crucial modality to make the system efficient. The fundamental frequency, energy, formats, mel-frequency cepstral coefficients (MFCC), and the Teager energy operator (TEO) are typical techniques used to capture stress and emotion features^34^. The identity vector (i-vector) and DNN embedding vector (x-vector) that have success in recognizing the speaker^35,36^ and language^37,38^ have also recently proven robust in representing the stress^13^ and emotion features^39^.

A single classifier, such as support vector machines (SVMs)^40,41^, neural networks and their variations^12,34^, the k-nearest neighbor (KNN), Gaussian mixtures model (GMM)^42^ and HMM^43^, is commonly used to discriminate the types of stress and emotions. To enhance the performance of single classifiers, hybrid classifiers such as SVM/GMM^44^ or ensemble models^11^ have been proposed. An amount of stress and emotion dataset (e.g., Speech Under Simulated and Actual Stress (SUSAS)^45,46^, Emotional Database (EmoDB)^47^, Keio University Japanese Emotional Speech Database (KeioESD)^48^, Ryerson Audio-Visual Database of Emotional Speech and Song (RAVDESS)^49^) has been provided. However, we know that stress has diverse characteristics and different patterns for each individual. It is caused by various aspects, such as characteristics, gender, experience background, and emotional tendencies^50^. Considering these rules, to make the system more robust and able to adapt in real conditions, more data training is required. Unfortunately, stress and emotion data are difficult to collect on a large scale.

To address this issue, some studies have explored an unsupervised approach for categorizing stress and emotion speech data based on the similarity of their characteristics. An unsupervised algorithm defines their effective objective in a self-learning manner^15–18,51,52^. Typically, an unsupervised clustering algorithm uses a similarity algorithm to compute the distance between data points in feature space^17,51,52^. However, calculating the distance for all data points on high-dimensional data is inefficient and known as the curse of dimensionality issue.

In the past year, some researchers have offered another approach for solving the problem of the curse of dimensionality by presenting a compact feature representation in the clustering assignment, known as deep clustering^53^. Deep clustering uses a DNN-based autoencoder to transform input into a low-dimensional feature representation and simultaneously learn the clustering assignment^20^. With this ability, deep clustering has become a popular clustering method and is widely used in many practical applications. Technically, deep clustering strengthens the feature representation by pushing the inter-cluster compactness. However, it accidentally ignores the effect of inter-cluster similarity. The unsupervised deep time-delay embedded clustering (DTEC)^21^ offers discriminative loss supervision to address this issue. DTEC has proven more effective in categorizing stress and emotions. Since DTEC is unsupervised learning, the correspondence between the output class and informational classes cannot be confirmed yet because there was no given measured information about the relationship between observed clusters. By incorporating prior knowledge, a semi-supervised DTEC framework (SDTEC)^22^ is proven to provide information for guiding the clustering assignment.

In some cases, emotion (e.g., stress) may change when triggered by an event while speaking^23^. Thus, we argue that the exploration of emotional state transition becomes a crucial consideration to recognize emotion accurately. Several studies explicitly modelled the speaker’s emotion by its state transition using KNN^23^, the long short-term memory (LSTM)^24^, Bayesian network^25^, finite state machine (FSM)^26^, and the Markov model^27^. Due to its ability to provide excellent representation for time-series (sequences) data^54,55^ with temporal variations^56^, the HMM is widely used to model the emotion state transition. A Markov model assumes that only the dependencies between consecutive hidden states are modeled so that there are local dependencies and limits for capturing a long-term temporal. To address this, the deep Markov neural network (DMNN) is proposed to learn in-depth the hidden representation of HMM using a recursive neural network^30^.

In this paper, the stress and emotion prediction model is proposed by considering its state transition. The proposed DTMN can learn in-depth the hidden representation of HMM using a fixed-dimension size of convolution networks (known as the time-delay neural network or TDNN). Different from DMNN that uses the recursive neural network to connect the previous time step of its hidden states, the proposed DTMN uses TDNN to model the relation between hidden states and the observations by receiving as input the activation patterns over time from units below. In addition, we apply a softmax function in the last layer to define the probability of each class. We evaluate the effectiveness of the DTMN to predict the stress and emotion state from the speech data of SUSAS^45,46^ and compare it with state-of-the-art state transition models, such as KNN^23^, LSTM^24^, the Bayesian network (BN)^25^, HMM^54^, and DMNN^30^. For further evaluation, we conducted an ablation experiment to investigate the effect of HMM and TDNN parameters on the prediction result.

## Results

We demonstrate the effectiveness of the proposed DTMN to predict the present state of stress and emotion and then model their state transition. The proposed DTMN is assigned to predict the state of stress and emotion from the speech data from the SUSAS dataset. The performance of DTMN is evaluated by comparing it with the baseline systems in terms of the prediction error rate (PER). Furthermore, we model the state transition of stress and emotions based on the speech label from the prediction result.

### Prediction accuracy

The effectiveness of the proposed DTMN is evaluated in predicting the emotional state of the time-series observations. In this experiment, we set the input and the parameters of DTMN as mentioned in the “DTMN parameters setting” section and the “Baseline systems setting” section, respectively. We run each system independently 10 times, and on average, the evaluation results are summarized in Table 1.

**Table 1:** The evaluation result of the proposed DTMN and the baseline systems in predicting the emotional state.

Method	Prediction error rate (% PER)
KNN^23^	48.27
BN^25^	41.63
HMM^54^	28.82
LSTM^24^	24.19
DMNN^30^	10.61
Proposed DTMN	8.55

Table 1 shows that BN presents a lower error than KNN. This is because KNN should provide proper scaling among variable time steps, while BN depicts the relationships between variables on each time step in the manner of conditional independencies. However, BN cannot represent the nonlinear functions of state variables. Hence, BN has a higher error rate than HMM. The performance gap between LSTM and HMM shows that in-depth learning of the hidden state is more effective than statistical machine learning. Although the LSTM has learned the long-term temporal context dependencies, many emotional states are hard to determine or even unobservable. The combination between HMM and DNN (such as DMNN and the proposed DTMN) presents a better ability in solving the LSTM’s limitations by demonstrating a lower error rate. By considering the activation patterns over time, the proposed DTMN significantly outperforms the DMNN in predicting the emotional state. The proposed DTMN is a sophisticated emotional state transition model that achieves an average prediction error rate of 8.55%.

### Emotional states transition

In the “Prediction accuracy” section, the proposed DTMN demonstrates an effective result in predicting the stress and emotion by its state transition. This indicates that the proposed DTMN can accurately predict the present state based on the prior states. Furthermore, we use a finite Markov chain to model the pattern of emotion transitions. Since males and females express emotion in different ways^57^, we present the state transition of males and females in the different diagrams.

**Figure 1:**
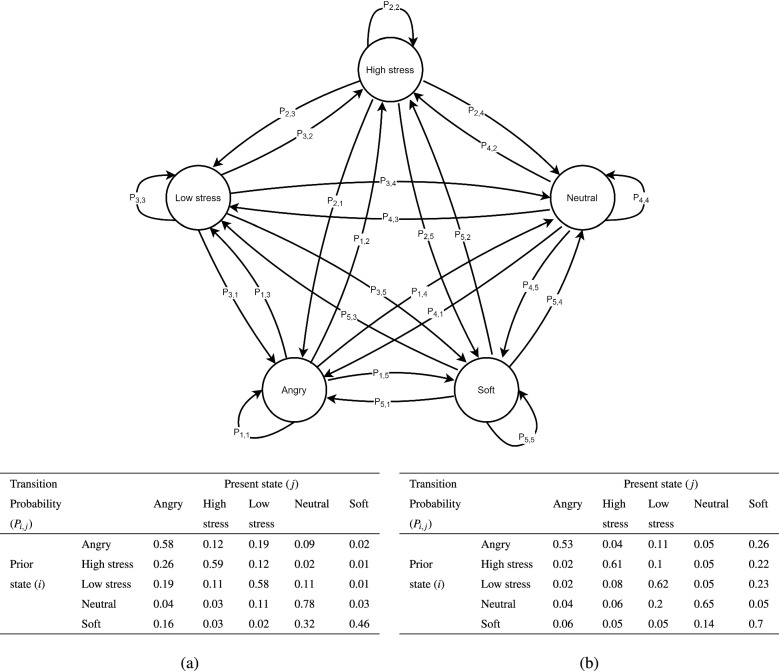
The state transition model of stress and emotions. Males and females present a similar emotional state transition model. Tables (**a**,**b**) show the transition probability from state *i* to state *j* for males and females, respectively.

Figure 1 shows the emotional state transition model. Tables (a) and (b) denote the state transition probability for males and females. $P_{i,j}$ indicates the transition probability from state *i* to states *j*. For instance, $P_{1,5}$ is the state transition probability from the state “angry” to state “soft” with the probability “0.02” for males and “0.26” for females. Each table shows that the sum of each row is one. As an example, the first row of Table (a) represents that sum of the transition probability from the state “angry” to the other states (angry, high stress, low stress, neutral, and soft) is one. This indicates that the transition matrix is a stochastic process, i.e., $\sum _{j} P(i,j)=1$. From Tables (a) and (b), it is clear that the highest probabilities of each row and column are diagonal. This indicates that emotions typically do not change in a short time. The current emotional state will be retained if there are no typical effective stimuli. However, the highest sum of each column is “neutral” for males and “soft” for females. This proves that females are more emotional than males. Another surprise is that females are more likely to be “soft”, while males are more likely to angry after stressful conditions, which indicates that gender responds to emotional stress in different reactions, both psychologically and biologically, depending on their background experience, behavioral, and physiological domains.

## Discussion

In this paper, we present a novel framework of stress and emotion prediction and modeling. Structurally, the DTMN consists of a HMM and the TDNN. The HMM is trained to produce the transition probabilities and the hidden states at each time step. TDNN can learn in-depth the hidden representation of HMM by creating more extensive networks from sub-components. In the prediction task, the DTMN is assigned to predict the emotional state of the time-series observations. As shown in Table 1, DTMN can outperform the baseline systems by achieving the lowest prediction error rate. This result indicates that the proposed DTMN overcomes the challenge by predicting the change in emotion accurately while speaking. Moreover, we showed that our method is efficient and effective in predicting stress and emotion.

As mentioned above, emotion can usefully be defined as states elicited by reinforcements. These reinforcements or stimuli can be considered emotional information. As we know, every person can recognize and understand other emotions without any training, and it is too complex to be described by machine learning. Therefore, we argue that there are common patterns of emotional events. In this work, we presume that the cognitive assessments to basic emotional stimuli are the same. Then, we use the five discrete emotional states (high stress, low stress, neutral, soft, and angry) from the SUSAS database and the movements of emotional states taken by the Markov process, as shown in Fig. 1. We represent males and females in different schemes because they express emotion in different ways. Generally, males and females present a similar emotional transition representation. However, there are some fundamental differences between male and female emotional transition tendencies. Females tend to more easily change their emotions, but they have a tendency to longer stress than males. After a stressful period, females tend to become “soft”, while males more easily become “angry”.

## Method

**Figure 2:**
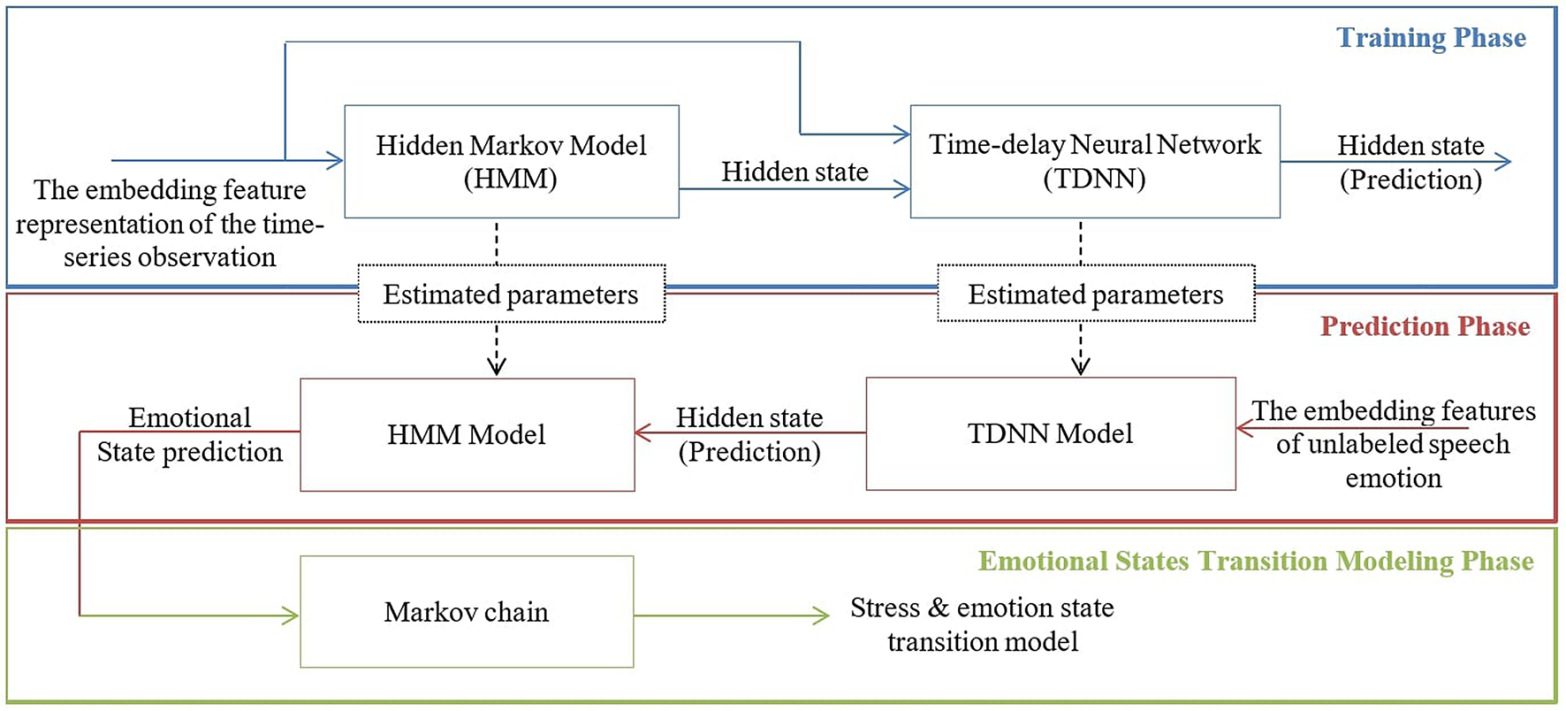
The framework for prediction and modeling the stress and emotions using the DTMN. The colored blue indicates the training phase, the color red denotes the prediction phase, and the colored green is the emotional states transition modeling phase.

The proposed DTMN structurally consists of a Markov model that is denoted by the HMM and a neural network that is represented by the TDNN. Figure 2 shows the framework for predicting and the stress and emotions using the proposed DTMN that is performed in three phases: the training phase, the prediction phase, and the emotional states transition modeling phase.

We perform a series of training procedures to obtain estimated parameters of DTMN. The HMM is trained using the time-series observation to produce the transition probabilities and the hidden states at each time step. Then, the TDNN is trained to predict the present hidden states using as input the present speech features and the prior hidden state. After the training phase, we obtain the estimated parameters of HMM and TDNN.

In the prediction phase, the trained DTMN is used to predict the emotional state label of the unlabeled observations. We conduct an opposite procedure with the training phase. First, the TDNN model predicts the present hidden states using the present speech features as input. Then, the HMM model predicts the emotional state label of the unlabeled observations using the predicted hidden states.

In the emotional states transition modeling phase, we model the transition pattern of emotions using the Markov chain with the predicted emotional states as input. This phase aims to illustrate the pattern of emotional state transitions of males and females. The Markov chain models five emotional states: high stress, low stress, neutral, soft, and angry.

### Deep time-delay Markov network

#### Hidden Markov model

The hidden Markov model (HMM) is a Markov chain whose internal state cannot be observed directly but only through some probabilistic function. In other words, the internal state of the model alone determines the probability distribution of the observed variables. This unobservable state is known as the hidden state. The advantage of the hidden states does not need to emphasize discretization and normalization issues so that we can deal with an arbitrary observation. In addition, the random noise in the observation can be handled by the hidden states. Therefore, the proposed DTMN uses the representation of the hidden states for connecting between observations.

**Figure 3:**
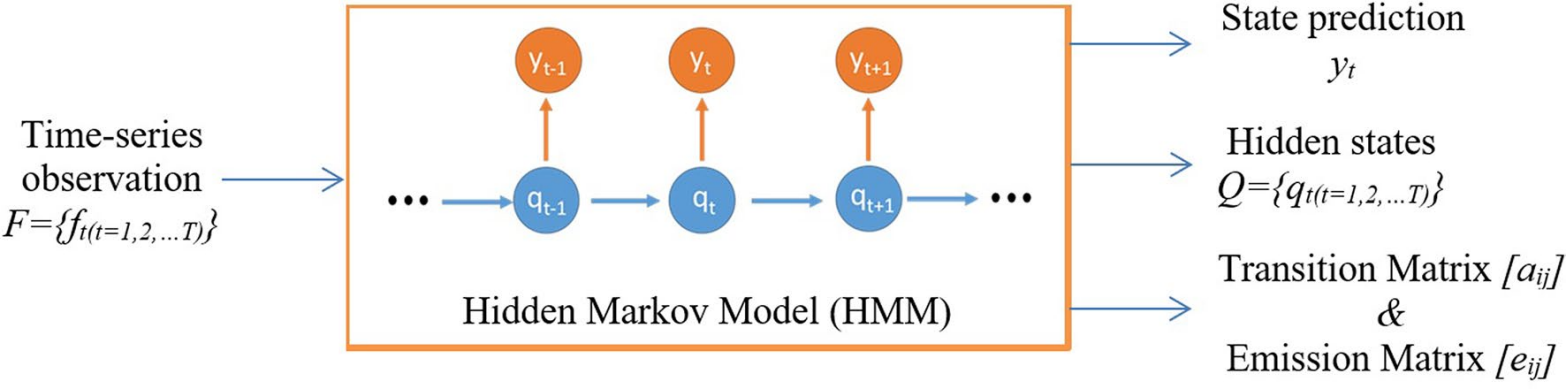
The hidden Markov model (HMM) training phase.

For instance, given an observation $f_t$ and a state label $y_t$, where $t=1,2\ldots ,T$. As shown in Fig. 3, $f_t$ and $y_t$ are the speech feature and the item that we want to predict at time *t*. By giving tuples $(f_t,y_t)$, a classification model is used to predict $y_t$. We present a hidden state variable $q_t$ on each time step to connect the observation $f_t$ and the label $y_t$. The parameter learning task in HMM is to find the best set of state transitions and emission probabilities. We establish the relationship between the hidden state and the labels as follows:

1 $$\begin{aligned} \begin{aligned} A=[a_{i,j}]&=P(q_t=i|q_{t-1}=j)\\ E=[e_{i,j}]&=P(y_t=i|q_t=j)\\ \end{aligned} \end{aligned}$$

where $i,j=\{1\ldots N\}$. Each $a_{ij}$ represents the probability of transition from state *i* to state *j*, and each $e_{ij}$ expresses the probability of $y_t$ being generated from state *j*.

#### Time-delay neural network

We use convolution networks with a fixed-dimension size (known as the time-delay neural network or TDNN) to predict the present hidden states. TDNN is a multilayer artificial neural network architecture that uses modular and incremental design to create more extensive networks from sub-components. It makes TDNN effective in learning the temporal dynamics of the signal even for short-term feature representation^31^. Unlike a standard DNN, in processing a wider temporal context, the first layer of TDNN learns the context in a narrow temporal window and continues to a deeper layer. Distinctively, TDNN receives input not only from the hidden state representation at the below layer but also from the activation pattern of the unit output and its context.

**Figure 4:**
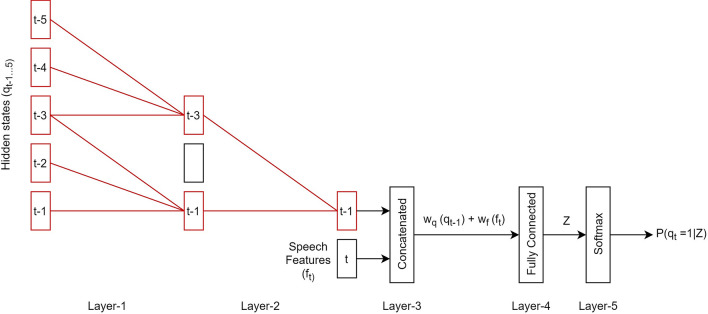
The structure of the TDNN.

In this paper, TDNN is used to model the relation between the hidden states and the observations by applying the relation of the hidden state and the labels (Eq. 1). Specifically, TDNN predicts the present hidden state $q_t$ by taking as input the prior hidden states $q_{t-1\ldots N}$ and the present features $f_t$. The structure of the TDNN is shown in Fig. 4, and each layer function is summarized in Table 2.

**Table 2:** The TDNN layer temporal context structure.

Layer	Feature context	Function
Layer-1	$[q_{t-5},q_{t-1}]$	Without sub-sampled
Layer-2	$\{q_{t-3},q_{t-1}\}$	Sub-sampled
Layer-3	$\{q_{t-1},f_t\}$	Concatenated
Layer-4	$\{0\}$	Fully connected
Layer-5	$\{0\}$	Softmax

As shown in Fig. 4 and Table 2, we designed a TDNN with five layers. Layer-1 holds full temporal contexts of prior hidden states from ${q_{t-5}}$ to ${q_{t-1}}$ that splices together frames $[0,-2]$. In Layer-2, we apply the sub-sampling technique (locally connected)^32^ so that only two temporal contexts (${q_{t-3}}$ and $q_{{t-1}}$) are held. Then, we concatenate the present speech features $f_t$ and $q_{t-1}$ feature from the second layer in Layer-3. A fully connected and softmax layer are performed in Layer-4 and Layer-5 of the TDNN, respectively. A softmax function is used to define the probability by taking a *C*-dimensional vector *Z* (from Layer-4) as input and outputs *C*-dimensional vector $\tau$ (real values between 0 and 1). The normalized exponential of the softmax function is expressed as follows:

2 $$\begin{aligned} \begin{aligned} \tau&=P(q_t=i|Z)=\frac{e^{Z_c}}{\sum _{d=1}^{C} e^{Z_d}} \quad {\textit{for }}d=1\ldots C \end{aligned} \end{aligned}$$

where $Z=w_{q}^{i},\alpha (q_{t-1})+w_{f}^{i}, \beta (f_t) + b$. $w_{q}$ and $w_f$ are the coefficients to be estimated. $\alpha$ and $\beta$ are the functions that are used to transform $q_{t-1}$ and $f_t$ into feature vectors. We perform a binary approach to $\alpha (q_{t-1})$ by assuming that the coordinates of $q_{t-1^{th}}=1$ and the others are zero. The denominator $\sum _{d=1}^{C} e^{z_d}$ is a regularizer that aims to ensure $\sum _{c=1}^{C} \tau =1$.

### Training phase

In the training phase, DTMN is trained to obtain the estimated parameters of HMM and TDNN. We perform the training phase in two steps. As shown in Fig. 2, the first step is to estimate the hidden state $q_t$ based on the labels $y_t$ using the Baum–Welch algorithm, and the transition matrix *A* and emission matrix *E* are estimated.

After $q_t$ is estimated, the second step is to estimate the parameter of the TDNN. We use the structure of the TDNN (Fig. 4) in the task of supervised prediction. The TDNN is trained to predict the hidden state $q_t$ on each time step. Iteratively, we estimate the TDNN’s parameters ($w_q$, $w_f$, and $\beta$) by minimizing the log-likelihood using stochastic gradient descent (SGD).

### Prediction phase

After the training phase, we obtain the estimated parameters of HMM (*A* and *E*) and TDNN’s parameters ($w_q$, $w_f$, and $\beta$). These estimated parameters are used to build the DTMN model.

In the prediction phase, we perform an opposite procedure with the training phase. The DTMN model is used to predict the label $y_t$ of the unlabeled observations using the present feature $f_t$ and prior hidden state $q_{t-1}$. By Eq. 2, we use $f_1$ to predict $q_t$, and then $q_1$ and $f_2$ are used to predict $q_2$. Next, to predict $q_3$, we used $(q_2,f_3)$. This procedure continues until $Q=\{q_{t,(t=1,2\ldots ,T)}\}$ are reached. Since each $q_t$ is a random variable and $P(q_t|f)$ is 1-by-1 from $t=1$ to $t=N$, the probability distribution of the labels $y_t$ that gives the prediction for the label is as follows:

3 $$\begin{aligned} \begin{aligned} P(y_t=i|f)&=\sum _{j}P(y_t=i|q_t=j).P(q_t=j|f)\\&=\sum _{j} e_{i,j}P(q_t=j|f) \end{aligned} \end{aligned}$$

### Emotional states transition modeling phase

A study^58^ defined emotions as discrete patterns of systemic activity. Emotions are categorized clearly and consistently across multiple levels of analysis, such as subjective experiences, physiological activity, and neural activation patterns. It supports that emotions are discrete systems that are organized in a distributed fashion across the brain.

A discrete system is characterized by a set of states and transitions between the states. To formally describe a discrete event simulation, many works use a stochastic process algebra^59,60^. In a discrete system, it can describe the passing of time and probabilistic choice between a limited number of processes, called the discrete stochastic process. Here, the universal quantifier is limited to feasible sequences of states to sequences that occur with positive probability. In other words, it is defined as a discrete stochastic process with a finite number of states.

Since emotions are discrete system activity^58^, we apply the finite Markov chain to model the state transitions of emotion. A finite set of states is high stress, low stress, neutral, soft, and angry. The emotional state updates its state depending on its current features and the prior states as input.

In this emotional state transition modeling phase, the state transition matrix *P* is represented by an $n \times n$ square Markov matrix in which each element is non-negative, and the sum of each row of *P* is one. Each row of *P* denotes a probability mass function for all *n* possible states. Given a finite set of state space *S* with *n* state value elements ${x_1,\ldots ,x_n}$. A Markov chain ${X_t}$ is a sequence of random variables on *S* that have the Markov property. This means that for any time step *t* and any state $y\in S$,

4 $$\begin{aligned} {\mathbb {P}}\{X_{t+1}=y|X_t\}={\mathbb {P}}\{X_{t+1}=y|X_t,X_{t-1\ldots }\} \end{aligned}$$

It indicates that probabilities for future states are known by just knowing the current state. Specifically, the set of values fully determines the dynamics of a Markov chain.

5 $$\begin{aligned} P(x,y):={\mathbb {P}}\{X_{t+1}=y|X_t=x\} \end{aligned}$$

where $(x,y) \in S$. With regard to *P*(*x*, *y*) being the transition probability from *x* to *y* in one step (time) and *P*(*x*.) being the conditional distribution of $X_{t+1}$ given $X_t=x$, *P* is obviously a stochastic matrix where:

6 $$\begin{aligned} P_{ij}=P(x_i,x_j) \end{aligned}$$

### Experiments

The experiments are conducted on a single personal computer with specifications: Intel Core i7-7700K CPU @ 4.2 GHz, 16 GB installed memory RAM, and a 64-bit operating system with an x64-based processor. For the software package, we used MATLAB software version R2017b^61^ with several toolboxes, such as deep learning, digital signal processing (DSP) systems, econometrics, audio, and signal processing.

#### Dataset

We used the stress speech data from the Speech Under Simulated and Actual Stress (SUSAS) databases that were collected by the Linguistic Data Consortium (LDC)^45^. The SUSAS database is divided into four domains of various stresses and emotions that were obtained from 32 speakers (13 women, 19 men)^46^. More than 16,000 utterances are provided in labeled and unlabeled data. SUSAS labels the speech data into five stress and emotion states: neutral, medium stress, high stress, soft, and angry. We used two labeled conversations data for estimating the two sets of parameters (HMM and TDNN). For evaluation, we used the six unlabeled conversations that have various speech durations.

We conditioned the speech input using their activity^62^, speakers^63^, and gender^64^. Then, each speech is represented in a low-dimensional embedding space using the SDTEC algorithm^22^.

#### DTMN parameters setting

In the HMM model, we set the number of hidden states to 80^30^, and the matrix of state transition and the initial state distribution are initialized randomly between 0 and 1. Gaussian distributions are used to determine the emission probabilities.

In the TDNN model, we perform batch normalization with a 256 batch size to stabilize the training procedure^30^. The rectified linear unit (ReLU) activation function is used on each hidden layer that has a dimension of 4000.

#### Baseline systems setting

The effectiveness of the proposed DTMN is evaluated to predict the stress and emotion state from the speech data of the SUSAS. We then compare it with five state-of-the-art state transition models, as follows:

KNN: run KNN with all parameter settings and architecture the same as^23^
BN: run the BN with all parameter settings and architecture as in^25^
HMM: run the HMM method with the same settings and architecture in^54^
LSTM: run the LSTM network with all parameter settings and architecture same as^24^
DMNN: run the DMNN with same setting and architecture in^30^

We use embedding feature representation from SDTEC (Section “Dataset”) as input to all systems (baseline and proposed system).

#### Ablation experiments

The ablation experiment is a method used to investigate the abilities of the system’s representations. It is especially helpful for observing the robustness of the system in an extensive work area^65^. The ablation experiment is an essential factor for safety-critical applications. Thus, to investigate the effectiveness of the proposed DTMN in more advanced applications, we conducted an ablation experiment. This experiment observes the effect of different values of the HMM and TDNN parameters on the prediction result. In particular, we analyze whether the number of hidden states (HMM model) and the number of temporal context inputs (TDNN model) are related to the prediction error rate (PER). In addition, we also observe the computational training time of the proposed DTMN compared to the baseline DMNN.

**Figure 5:**
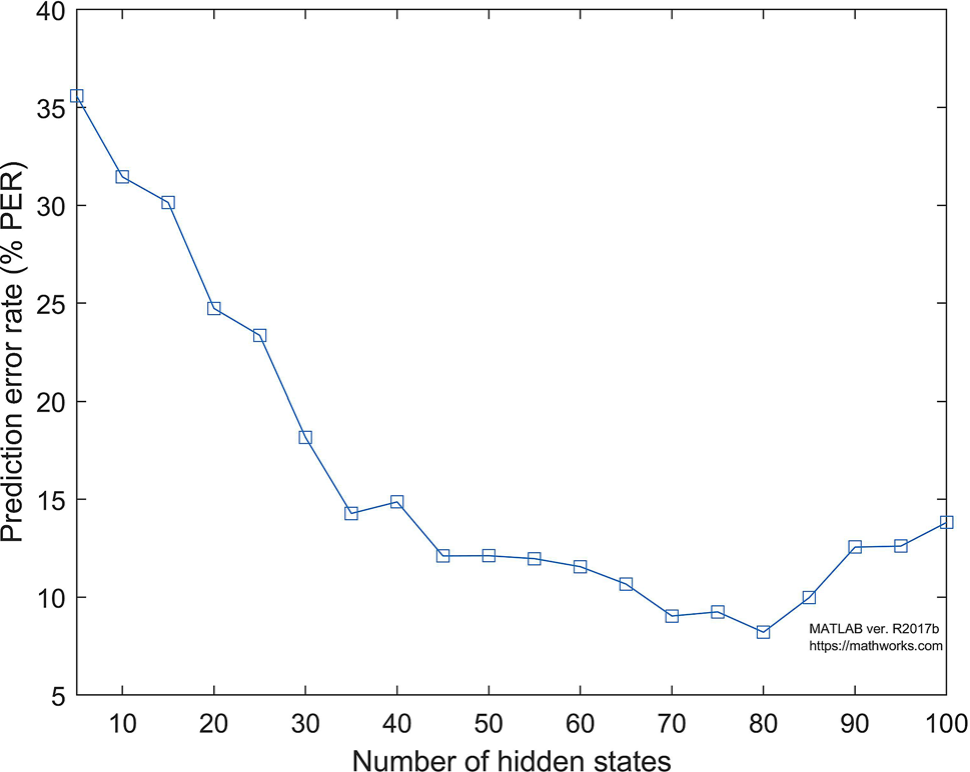
The effect of the number of hidden states in the prediction result.

We estimate the hidden states $q_t$ based on the labels $y_t$ using the Baum-Welch algorithm. Additionally, the estimated state transition matrix *A* and emission matrix *E* are obtained, as expressed in Eq. (1). Specifically, the Baum-Welch algorithm uses the expectation-maximization (EM) algorithm to find the maximum likelihood estimate of the parameters of the hidden Markov model (HMM) given a set of observed feature vectors. The maximum likelihood approach can produce an HMM that significantly overfits the limit and consequently exaggerates the number of hidden states present in the signal. Hence, we argue that a correct selection of the number of hidden states in the HMM context is a crucial problem that should be observed. In this experiment, we run the HMM model by setting a different number of hidden states (5–100). Figure 5 shows the prediction error rate in different numbers of hidden states. It shows that the increase in the number of hidden states reduces the prediction error rate significantly. The lowest error rate is achieved when the number of hidden states is 80.

Because each process in the TDNN architecture is bound to the time steps, they look like the convolutional network. An accumulated gradient updates the lower-layer hyperparameters across input time steps. TDNN computes the activation of the time steps at each layer and the dependencies across layers. Hence, a correct temporal contextual input determines the effectiveness of the TDNN architecture. Thus, in this section, we investigate the effectiveness of the TDNN with various temporal contexts on the prediction result. We set each neural network to have 4000-dimensional input. The investigation of the various temporal contexts is conducted on the first two layers of the TDNN architecture (Layer-1 and Layer-2), see Fig. 4.

TDNN predicts the present hidden state by using as input a set of the prior hidden states $q_{t-1\ldots T}$ from the HMM. The prediction error rate of the TDNN with various temporal context inputs is demonstrated in Table 3. TDNN-1 presents the highest error prediction compared to the other models. This indicates that multi-temporal context input is better for predicting present emotional state than a single temporal context. Furthermore, the increase in the number of temporal contexts (TDNN-2 and TDNN-3) can decrease the prediction error rate significantly. TDNN-4, which uses $[-1,-5]$ as input, is the optimal temporal context for predicting the emotional state. It achieves 8.31% PER.

**Table 3:** The performance comparison of TDNN with various temporal contexts.

Model	Network context	Layerwise context			PER (%)
		1	2	3	
TDNN-1	$\{-1\}$	$\{-1\}$	$\{-1\}$	$\{-1\}$	10.08
TDNN-2	$\{-1,-2\}$	$\{-1,-2\}$	$\{-1\}$	$\{-1\}$	9.76
TDNN-3	$[-1,-3]$	$\{-1,-2\}$	$\{-1,-2\}$	$\{-1\}$	9.02
TDNN-4	$[-1,-5]$	$[-1,-3]$	$\{-1,-3\}$	$\{-1\}$	8.31
TDNN-5	$[-1,-7]$	$[-1,-3]$	$\{-1,-3,-5\}$	$\{-1\}$	8.79
TDNN-6	$[-1,-9]$	$[-1,-5]$	$\{-1,-5,-9\}$	$\{-1\}$	8.80

**Figure 6:**
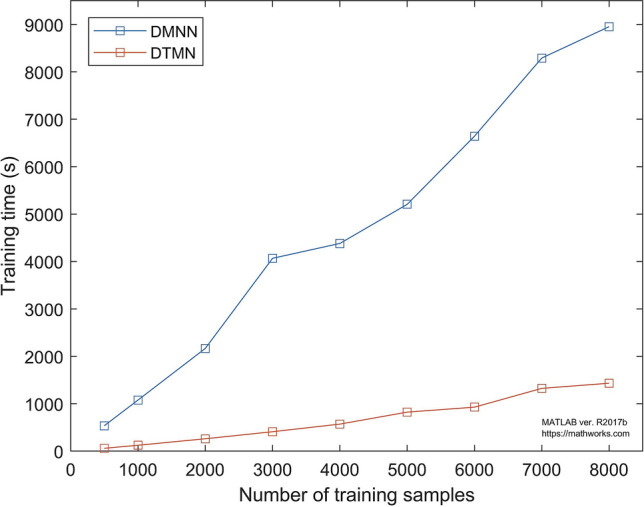
The computational training time of the proposed DTMN and the baseline DMNN for different numbers of training samples.

The proposed DTMN models the temporal dynamics by capturing the long-term dependencies between states. Hence, it requires an acoustic model that can effectively deal with long temporal contexts. In the “Prediction accuracy” section, the effectiveness in modeling the temporal dynamics of the DTMN is evaluated in terms of the prediction error rate (PER). The accuracy of the prediction result is essential, but in practice (implementation phase), the time complexity of the model should also be considered. Training involves finding a specific set of weights based on training examples, which yields a predictor that has excellent performance. Thus, training time is the main challenge in developing a model. Existing theoretical results show that a model that is computationally difficult is the worst model^66^. Hence, in this ablation experiment, we observe the training time of the proposed DTMN, presented in Fig. 6. We demonstrate the computational training time of the proposed DTMN compared to the baseline DMNN in different numbers of training samples (from 500 to 8,000). In this experiment, we train the systems on a computer with specifications, as mentioned in the “Experiments” section. Figure 6 shows that DTMN presents a lower computational training time than DMNN (1,433 seconds for DTMN and 8,952 seconds for DMNN in 8,000 training samples). As mentioned before, DTMN uses TDNN to model the relation between hidden states and observations. TDNN operates at a different temporal resolution, which increases on higher layers of the network. The transforms in the TDNN are tied across time steps, and for this reason, the lower layers of the network can learn invariant feature transforms effectively. Moreover, as shown in Fig. 4, we applied the sub-sampled technique. This technique makes the computations of the time step activations more efficient than standard DNN.

## Conclusion

In this paper, we proposed a new framework for predicting and modeling stress and emotions, named the deep time-delay Markov network (DTMN). DTMN predicted the state of stress and emotions by considering its state transition. Structurally, the proposed DTMN consisted of a hidden Markov model (HMM) and the time-delay neural network or TDNN. HMM was used to predict the hidden states at each time step, while the neural network was applied to learn in-depth the hidden representation of HMM. The TDNN predicts the present hidden state using as input the prior hidden states and the features of the present time. We explicitly used a compact feature representation of stress and emotion (embedding features) of SDTEC as the input of DTMN. The effectiveness of the proposed DTMN was evaluated by comparing it with some state transition models, such as KNN, LSTM, the Bayesian network, HMM, and DMNN, in the task of predicting the emotional state from the time-series data of the SUSAS dataset. Based on the evaluation result, the proposed DTMN outperformed the baseline state transition systems by achieving a prediction error rate (PER) of 8.55%. In further analysis, we conducted a comprehensive ablation experiment to investigate whether the estimated parameters of HMM and TDNN are related to model performance. In particular, we investigated a different number of hidden states in the HMM and the various temporal contexts in the TDNN parameters to the prediction result and the computational training time of the proposed DTMN. The experimental results showed that the lowest error rate was achieved for the number of hidden states by 80, the temporal context of TDNN is $[t-1,t-5]$, and the computational training time of the DTMN is 1,400 seconds for 8,000 training samples. Furthermore, we performed a finite Markov chain to model the state transition of stress and emotions. Based on the emotional state transition model, females have a trend in longer stress conditions than males. After a stressful period, females have a probability to be more easily soft, while males tend more easily to anger. In general, females are more emotional than males.

Non-intrusive measurement methods (such as facial or speech) are not as effective as non-invasive methods (such as EEG and ECG). However, based on the experimental results, the proposed method presented a low error rate in recognizing stress and emotions. In other words, the proposed system demonstrates great promise to be leveraged in real life. Therefore, in the future, we will implement a smart-phone application-based proposed system as an early detection system of emotion.
